# Supplementary material for: Long-range optical coupling with epsilon-near-zero materials
Source: Nat Commun. 2025 Oct 16;16:9172. doi: 10.1038/s41467-025-64504-w (PMC12531341; doi:10.1038/s41467-025-64504-w)
Supplement: Supplementary file 1 — Supplementary Information [file 41467_2025_64504_MOESM1_ESM.pdf]

SUPPLEMENTAL INFORMATION

Long-range Optical Coupling with Epsilon-near-zero Materials

Danqing Wang[1,2,3,4]*, Zheyu Lu[4,5,6], Sorren Warkander[2], Niharika Gupta[7], Qingjun Wang[2], Penghong Ci[2,8], Ruihan Guo[2,4], Jiachen Li[2], Ali Javey[4,7], Jie Yao[2,4], Feng Wang[4,5], Junqiao Wu[2,4]*

[1]College of Future Information Technology, Shanghai Engineering Research Centre of Ultra-precision Optical Manufacturing, Fudan University, Shanghai 200433, China

[2]Department of Materials Science and Engineering, University of California, Berkeley, Berkeley, CA 94720, USA

[3]Miller Institute for Basic Research in Science, University of California, Berkeley, Berkeley, CA 94720, USA

[4]Materials Sciences Division, Lawrence Berkeley National Laboratory, Berkeley, CA 94720, USA

[5]Department of Physics, University of California, Berkeley, Berkeley, CA 94720, USA

[6]Graduate Group in Applied Science and Technology, University of California, Berkeley, Berkeley, CA 94720, USA

[7]Department of Electrical Engineering, University of California, Berkeley, Berkeley, CA 94720, USA

[8]State Key Laboratory of Semiconductor Physics and Chip Technologies, Institute of Semiconductors, Chinese Academy of Sciences, Beijing 100083, China

*Corresponding author: danqingwang@fudan.edu.cn; wuj@berkeley.edu

Supplementary Note S3

Supplementary Fig. 1. Optical resonance at the ENZ wavelength at oblique incidence under transverse magnetic polarization S7

Supplementary Fig. 2. Strong optical fields at sub-wavelengths in ENZ thin films S8

Supplementary Fig. 3. Analogy to resonant tunneling in quantum double barriers S9

Supplementary Fig. 4. Correlated optical field intensity between ENZ layers S10

Supplementary Fig. 5. Evanescent near-field coupling at submicrons in double metal thin films S11

Supplementary Fig. 6. Near-field oscillations in low-index dielectric multilayers S13

Supplementary Fig. 7. ENZ multilayers for long-distance optical coupling S14

Supplementary Fig. 8. Long-distance coupling between ENZ thin films up to hundreds of microns S15

Supplementary Fig. 9. Sustained long-distance coupling up to hundreds of microns despite materials loss S16

Supplementary Fig. 10. Wavelength as an additional degree of freedom in ENZ systems S17

Supplementary Fig. 11. Angled sputtering deposition for fabricating wedged ITO thin films S18

Supplementary Fig. 12. Separation-dependent transmission spectra showing the oscillatory ENZ resonances... S19

Supplementary Fig. 13. Optical setup scheme for the nonlinear optics measurements..... S21

Supplementary Fig. 14. Measured SHG from a single-layer ITO thin film....................... S22

Supplementary Fig. 15. Modulated SHG intensity by ENZ resonances in a single-layer ITO film.. S23

Supplementary Fig. 16. Modeled SHG with finite-difference time-domain method.......... S24

Supplementary Fig. 17. Fitting of ITO permittivity characterized by ellipsometry S26

Supplementary Fig. 18. Mathematical model for modulated SHG by total internal reflection S27

Supplementary References... S28

Supplementary Note

Resonant tunneling through a single quantum barrier

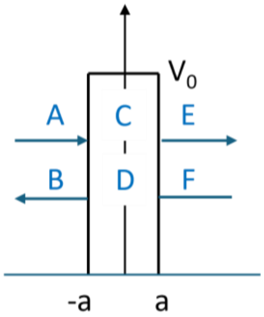

As the above figure, we consider a single quantum barrier in one dimension, whose height is V_0 in the region $|x| < a$ (the derivations here are taken from ref. 53). Outside of this barrier, the particle can exist as a freely propagating wave. In the region $|x| < a$, and for energies $\mathcal{E} < V_0$, the wave is heavily attenuated and is characterized by a decaying evanescent wave.
We can define the wave vector k in the region $|x| > a$, and the decaying wave vector γ in the region $|x| < a$,

$$k = \sqrt{\frac{2m}{\hbar^2}\mathcal{E}} \quad \gamma = \sqrt{\frac{2m}{\hbar^2}(V_0 - \mathcal{E})} \tag{1}$$

Thus, we can write the wave function as

$$\Psi(x) = \begin{cases} A\mathrm{e}^{\mathrm{i}kx} + B\mathrm{e}^{-\mathrm{i}kx} & x < -a \\ C\mathrm{e}^{\gamma x} + D\mathrm{e}^{-\gamma x} & |x| < a \\ E\mathrm{e}^{\mathrm{i}kx} + F\mathrm{e}^{-\mathrm{i}kx} & x > a \end{cases} \tag{2}$$

The boundary conditions are applied by matching the continuity of the wave function and its derivative at the interfaces. At $x = -a$,

$$A\mathrm{e}^{-\mathrm{i}ka} + B\mathrm{e}^{\mathrm{i}ka} = C\mathrm{e}^{-\gamma a} + D\mathrm{e}^{\gamma a} \tag{3a}$$
$$\mathrm{i}k\left[A\mathrm{e}^{-\mathrm{i}ka} - B\mathrm{e}^{\mathrm{i}ka}\right] = \gamma[C\mathrm{e}^{-\gamma a} - D\mathrm{e}^{\gamma a}]. \tag{3b}$$

This leads to the transmission matrix equation

$$\begin{bmatrix} A \\ B \end{bmatrix} = \left[\begin{bmatrix} \left(\frac{\mathrm{i}k + \gamma}{2\mathrm{i}k}\right)\mathrm{e}^{(\mathrm{i}k-\gamma)a} & \left(\frac{\mathrm{i}k - \gamma}{2\mathrm{i}k}\right)\mathrm{e}^{(\mathrm{i}k+\gamma)a} \\ \left(\frac{\mathrm{i}k - \gamma}{2\mathrm{i}k}\right)\mathrm{e}^{-(\mathrm{i}k+\gamma)a} & \left(\frac{\mathrm{i}k + \gamma}{2\mathrm{i}k}\right)\mathrm{e}^{-(\mathrm{i}k-\gamma)a} \end{bmatrix}\begin{bmatrix} C \\ D \end{bmatrix}\right]. \tag{4}$$

At $x = a$,

$$E\mathrm{e}^{\mathrm{i}ka} + F\mathrm{e}^{-\mathrm{i}ka} = C\mathrm{e}^{\gamma a} + D\mathrm{e}^{-\gamma a} \tag{5a}$$
$$\mathrm{i}k\left[E\mathrm{e}^{\mathrm{i}ka} - F\mathrm{e}^{-\mathrm{i}ka}\right] = \gamma[C\mathrm{e}^{\gamma a} - D\mathrm{e}^{-\gamma a}]. \tag{5b}$$

Again, this leads to

$$\begin{bmatrix} C \\ D \end{bmatrix} = \begin{bmatrix} \left(\frac{\mathrm{i}k+\gamma}{2\gamma}\right) \mathrm{e}^{(\mathrm{i}k-\gamma)a} & -\left(\frac{\mathrm{i}k-\gamma}{2\gamma}\right) \mathrm{e}^{-(\mathrm{i}k+\gamma)a} \\ -\left(\frac{\mathrm{i}k-\gamma}{2\gamma}\right) \mathrm{e}^{(\mathrm{i}k+\gamma)a} & \left(\frac{\mathrm{i}k+\gamma}{2\gamma}\right) \mathrm{e}^{-(\mathrm{i}k-\gamma)a} \end{bmatrix} \begin{bmatrix} E \\ F \end{bmatrix} \tag{6}$$

Together, we can write

$$\begin{bmatrix} A \\ B \end{bmatrix} = \begin{bmatrix} M_{11} & M_{12} \\ M_{21} & M_{22} \end{bmatrix} \begin{bmatrix} E \\ F \end{bmatrix} \tag{7}$$

Here, the elements are defined by

$$\begin{aligned} M_{11} &= \left(\frac{\mathrm{i}k+\gamma}{2\mathrm{i}k}\right)\left(\frac{\mathrm{i}k+\gamma}{2\gamma}\right) \mathrm{e}^{2(\mathrm{i}k-\gamma)a} - \left(\frac{\mathrm{i}k-\gamma}{2\gamma}\right)\left(\frac{\mathrm{i}k-\gamma}{2\mathrm{i}k}\right) \mathrm{e}^{2(\mathrm{i}k+\gamma)a} \\ &= \left[\cosh(2\gamma a) - \frac{\mathrm{i}}{2}\left(\frac{k^2-\gamma^2}{k\gamma}\right) \sinh{(2\gamma a)}\right] e^{2ika} \end{aligned} \tag{8}$$

$$M_{21} = \left(\frac{\mathrm{i}k+\gamma}{2\gamma}\right)\left(\frac{\mathrm{i}k-\gamma}{2\mathrm{i}k}\right) \mathrm{e}^{-2\gamma a} - \left(\frac{\mathrm{i}k+\gamma}{2\mathrm{i}k}\right)\left(\frac{\mathrm{i}k-\gamma}{2\gamma}\right) \mathrm{e}^{2\gamma a}$$

$$= -\frac{\mathrm{i}}{2}\left(\frac{k^2+\gamma^2}{k\gamma}\right) \sinh{(2\gamma a)} \tag{9}$$

$$M_{22} = M_{11}^* \quad M_{12} = M_{21}^* \tag{10}$$

Since we treat an incoming wave from only the left side of the two sides, so $F = 0$. Then, we find that $A = M_{11}E$. The transmission probability is

$$T = \frac{1}{|M_{11}|^2} \tag{11}$$

Combining the value from (8), we find

$$T\ (\mathcal{E} < V_0) = \left[\cosh^2{(2\gamma a)} + \left(\frac{k^2-\gamma^2}{2k\gamma}\right)^2 \sinh^2{(2\gamma a)}\right]^{-1} \tag{12}$$

Notably, for incident energy above the barrier ($\mathcal{E} > V_0$), the transmission probability can be derived by making the substitution $\gamma \rightarrow -\mathrm{i}k'$, where

$$k' = \sqrt{\frac{2m}{\hbar^2}(\mathcal{E} - V_0)} \tag{13}$$

This changes (12) into

$$T(\mathcal{E} > V_0) = \frac{1}{1 + \left(\frac{k^2 - k'^2}{2kk'}\right)^2 \sin^2{(2k'a)}} \tag{14}$$

For $\mathcal{E} > V_0$, the transmission shows oscillatory behavior as a function of energy, with unity transmission that occurs for $\boldsymbol{2k'a = m\pi}$, where m is an integer. Thus, resonant tunneling for a single quantum barrier happens when **constructive interference** occurs inside the barrier, which is determined by the barrier thickness $2a$, incident particle energy $\mathcal{E}$, and barrier potential V_0.

Resonant tunneling through quantum double barriers

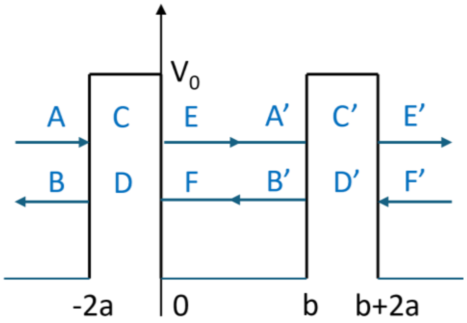

We now study two tunnel barriers separated by a quantum well in one dimension[1,2]. The tunneling transmission coefficient can be solved by combining the transmission matrices of two barriers. We note that both E and A' describe a wave propagating to the right. Denoting the thickness of the well region as b, we can relate these two coefficients via

$$A' = E\mathrm{e}^{\mathrm{i}kb} \tag{15}$$

where k is the propagation constant in the well region. Similarly, F and B' describe the same wave propagating in the opposite direction.

$$B' = F\mathrm{e}^{-\mathrm{i}kb} \tag{16}$$

Thus,

$$\begin{bmatrix} E \\ F \end{bmatrix} = \begin{bmatrix} \mathrm{e}^{-\mathrm{i}kb} & 0 \\ 0 & \mathrm{e}^{+\mathrm{i}kb} \end{bmatrix} \begin{bmatrix} A' \\ B' \end{bmatrix} \tag{17}$$

Equation (17) now defines a matrix $\mathbf{M}_\mathrm{W}$, where the subscript indicates the well region. The overall tunneling matrix can be written as

$$\begin{bmatrix} A \\ B \end{bmatrix} = [\mathbf{M}_\mathrm{L}][\mathbf{M}_\mathrm{W}][\mathbf{M}_\mathrm{R}]\begin{bmatrix} E' \\ F' \end{bmatrix} \tag{18}$$

From this, the total composite $M_{\mathrm{T}11}$ is written as

$$M_{\mathrm{T}11} = M_{\mathrm{L}11}M_{\mathrm{R}11}\mathrm{e}^{-\mathrm{i}kb} + M_{\mathrm{L}12}M_{\mathrm{R}21}\mathrm{e}^{\mathrm{i}kb} \tag{19}$$

Here, we assume that the two barriers are equal, so the same propagation wave vector k exists in the well and the regions to the left and right of the double barriers. In order to simplify the mathematical details, we write (8) as

$$M_{11} = m_{11}\mathrm{e}^{-\mathrm{i}\theta} \tag{20}$$

where

$$m_{11} = \sqrt{\cosh^2(2\gamma a) + \left(\frac{k^2 - \gamma^2}{2k\gamma}\right)^2 \sinh^2(2\gamma a)} \tag{21}$$

is the magnitude, and

$$\theta = \tan^{-1}\left[\left(\frac{k^2 - \gamma^2}{2k\gamma}\right)\tanh(2\gamma a)\right] \tag{22}$$

is the phase of M_{11}. We then use this to write

$$|M_{\mathrm{T}11}|^2 = |M_{11}|^4 + |M_{21}|^4 + 2|M_{11}|^2|M_{21}|^2\cos[2(kb+\theta)]$$
$$= (|M_{11}|^2 - |M_{21}|^2)^2 + 4|M_{11}|^2|M_{21}|^2 cos^2(kb+\theta)$$

The first term is the determinant of the individual barrier matrix and is thus unity for a simple rectangular barrier. The overall transmission is

$$T_{\mathrm{total}} = |M_{\mathrm{T}11}|^{-2} = [1 + 4|M_{11}|^2|M_{21}|^2\cos^2(kb+\theta)]^{-1} \tag{23}$$

The above cosine term vanishes and $T_{\mathrm{total}} = 1$, when

$$\boldsymbol{kb + \theta = (2m+1)\frac{\pi}{2}} \tag{24}$$

which is the resonance condition for quantum tunneling in double barriers. The resonant tunneling for quantum double barriers is determined by the barrier distance d, barrier thickness $2a$, incident particle energy $\mathcal{E}$, and barrier potential V_0.

In sum, as a side-by-side comparison between the quantum double barriers and epsilon-near-zero (ENZ) bilayers, the wavefunction can propagate through the quantum wells, resembling the electromagnetic wave propagation through the glass substrate as well as the silica spacer. In addition, the low-dispersive ENZ modes within ENZ layers sustain a nearly zero group velocity and nearly infinite group index. Hence, the induced evanescent wave decay within ENZ thin films is analogous to the evanescent wavefunction trapped within quantum double barriers.

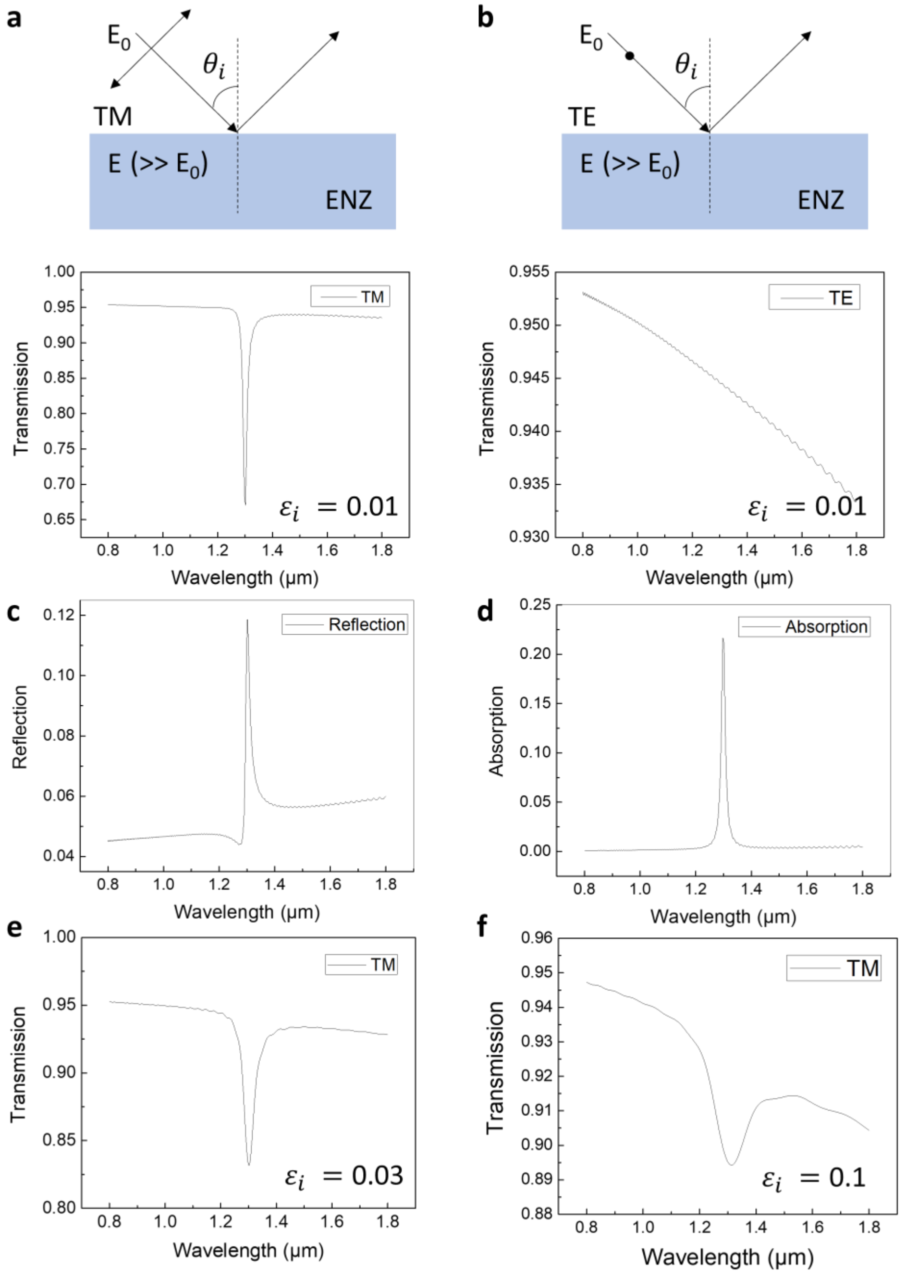

Supplementary Fig. 1. Optical resonance at the ENZ wavelength at oblique incidence under transverse magnetic polarization. (**a**) Transmission spectrum of an ENZ thin film (imaginary part of permittivity ε_i = 0.01) under transverse magnetic (TM) polarization. (**b**) Transmission spectrum of an ENZ thin film under transverse electric (TE) polarization. The film thickness is d = 50 nm, the incident angle is θ_i = 4°, and the plotting wavelength is λ = 1.3 μm. The decay curve under TE polarization represents increased material loss at longer optical frequencies in a Drude model system. (**c-d**) Reflection and absorption spectra at the ENZ wavelength. The optical extinction at ENZ wavelength is partly from light reflection (35%) and partly from light absorption (65%), the latter indicating trapped light within ENZ thin film as attributed to Berreman resonance[48]. (**e**) Transmission spectrum of an ENZ thin film with ε_i = 0.03. (**f**) Transmission spectrum of an ENZ thin film with ε_i = 0.1.

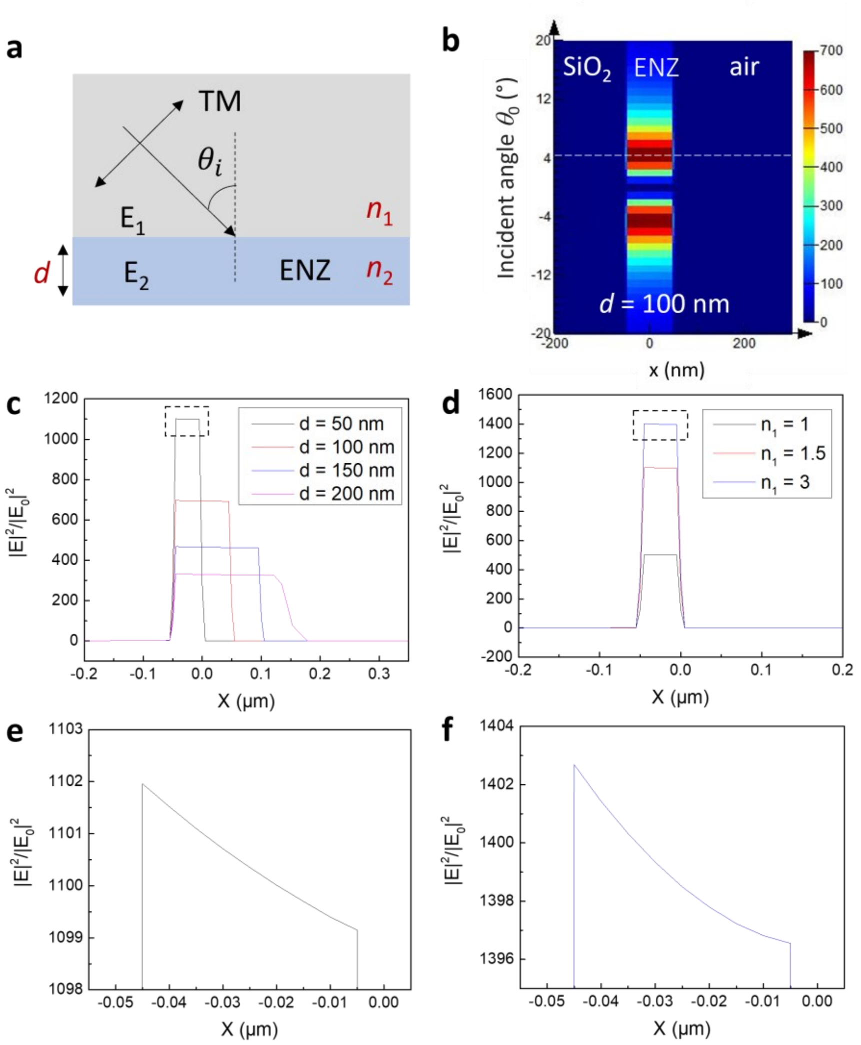

Supplementary Fig. 2. Strong optical fields at subwavelength scales in ENZ thin films. (**a**) Scheme of numerical modeling with finite-difference time-domain method. (**b**) Angle-resolved near-field distribution for a single ENZ thin film (thickness d = 100 nm). (**c**) Stronger optical near fields were observed for thinner ENZ films. Refractive index of the incident plane medium is n_1 = 1.5. (**d**) Stronger near fields were observed for ENZ films with better index contrast at the ENZ/dielectric interface. Film thickness is d = 50 nm. (**e-f**) Zoom-in plots for the spatial distribution of near-field intensity in the dashed regions of panels c and d, respectively. These plots showed evanescent decay of optical near fields over distance within ENZ layers, which is consistent with Fig. 1b. The continuity of the electric displacement field D is essential in producing the electric field enhancement ($\nabla \cdot D = \rho_{e0}$, $D_{2\perp}$ - $D_{1\perp}$ = ρ_{e0} = 0, if no charge exists at the interface). Hence, $\varepsilon_0 E_{1\perp} = \varepsilon_0 \varepsilon E_{2\perp}$. For ENZ materials, where ε = 0 and $E_{1\perp}$ is a nonzero number (at oblique angle incidence), the normal component of the E field $E_{2\perp}$ is strongly enhanced within the film. In contrast, at normal incidence (θ_i = 0°), $E_{1\perp}$ = 0, which also drives $E_{2\perp}$ to be zero, and hence, no strong field enhancement exists.

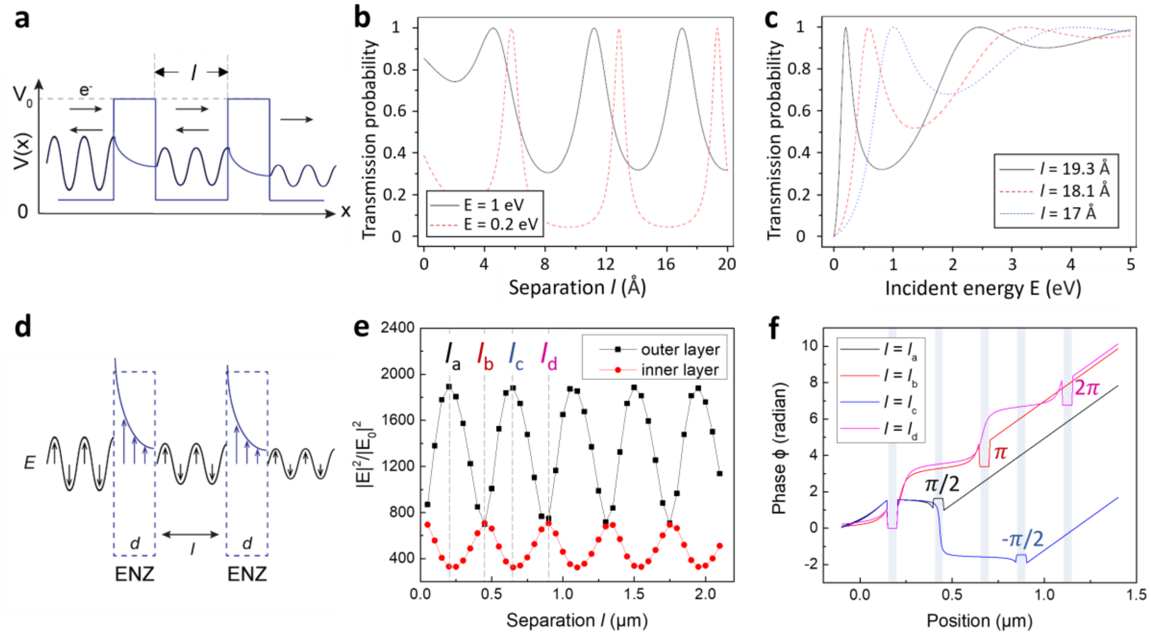

Supplementary Fig. 3. Analogy of optical coupling in ENZ bilayers to resonant tunneling in quantum double barriers. (**a**) Scheme of resonant tunneling in quantum double barriers (E < V_0). (**b**) Variations of the transmission probability with energy for several barrier separation distances *l*. (**c**) Transmission probability as a function of barrier distance *l* at fixed incident energy. Panels b-c adapted from ref. 3 with permission; copyright 2018 Springer. In equal quantum double barriers, resonant tunneling with a unity transmission happens when constructive interference happens at the well between two barriers, which is sensitive to the incident particle energy E and barrier separation distance *l*. (**d**) Scheme of an optical system composed of two ENZ thin films separated by a dielectric spacer. Analogous to quantum double barriers, the glass substrate and dielectric spacer in ENZ bilayers serve as the transmission media, and the ENZ layer functions as the barrier for light propagation. (**e**) Near-field oscillations for the ENZ/silica/ENZ multilayers on silica substrate under TM polarization. (**f**) Phase distribution for the ENZ/silica/ENZ multilayers at different interlayer separation distances (l_a, l_b, l_c, and l_d) in panel e. The blue regions suggest the position of two ENZ layers in each modeling. ENZ thin films showed nearly-zero phase shift within a single layer, and correlated phase difference between two films as determined by the separation distance *l*. For $l = l_a$, l_b, l_c, and l_d in panel e, the phase difference between the inner and outer ENZ layer ($\Delta\phi = \phi_{inner} - \phi_{outer}$) is π/2, π, - π/2, and 2π, respectively, which corresponds to constructive (l_b, l_d) and destructive interference (l_a, l_c) between two ENZ layers. As analogous to resonant tunneling in double barriers that show unity transmission under constructive interference, the inner ENZ layer's optical field intensity reaches a local maximum when constructive interference occurs between two layers with a phase difference of *m*π, where *m* is an integer. In sum, the analogy between ENZ bilayers and quantum double barriers is based on their similar wavefunction distribution (Figure 1b) as well as the same interaction mechanism, as tuned by phase in both cases.

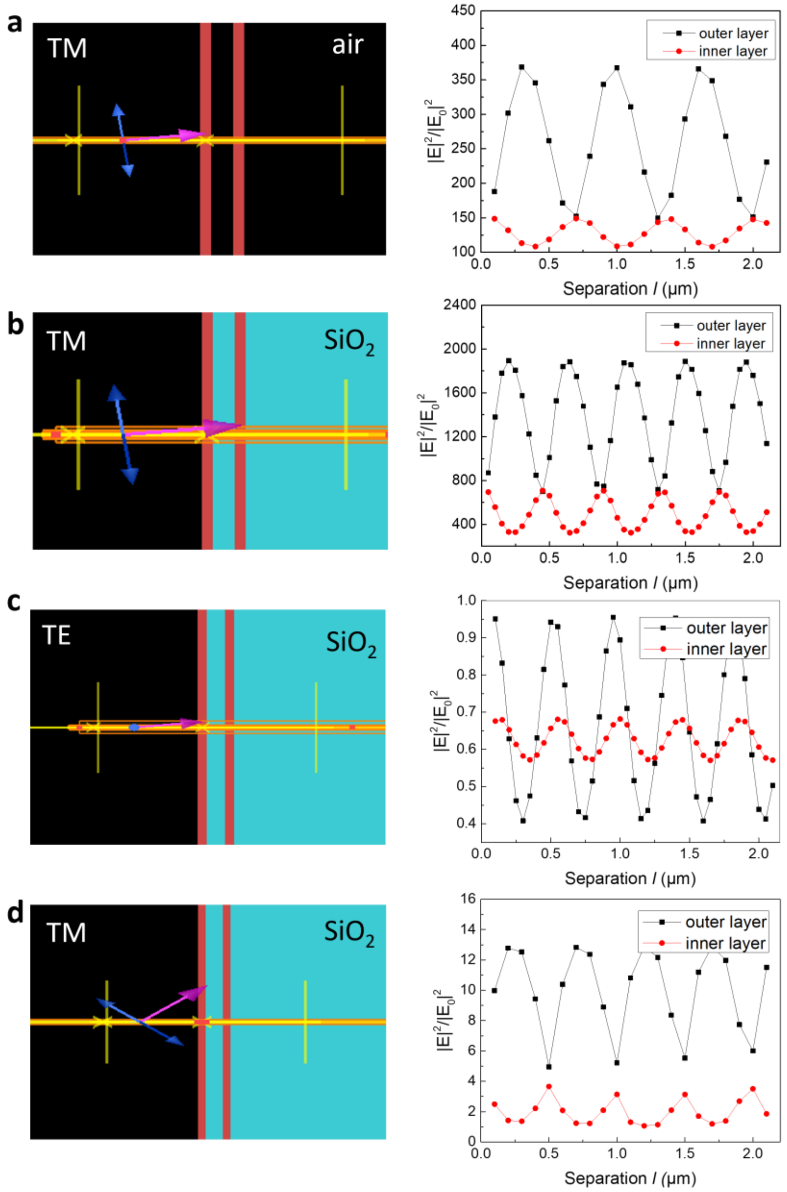

Supplementary Fig. 4. Correlated optical field intensity between ENZ layers. (**a**) Near-field oscillations for the ENZ/air/ENZ multilayers under TM polarization. (**b**) Near-field oscillations for the ENZ/silica/ENZ multilayers on silica substrate under TM polarization. (**c**) Near-field oscillations for the ENZ/silica/ENZ multilayers on a silica substrate under TE polarization. Optical field intensity in the outer and inner ENZ layers showed anti-correlated oscillations under TM polarization. In contrast, weak thin-film interference occurred under TE polarization with uncorrelated optical near fields between the outer and inner layers. Incident angle is θ_i = 7°, and the pump wavelength is λ = 1.3 μm. (**d**) Near-field oscillations for the ENZ/silica/ENZ multilayers with materials loss ε_i = 0.1 in the modeling for ENZ layers and at an incident angle θ_i = 45°. With materials loss included, the long-range coupling with anti-correlated near fields is sustained for the ENZ bilayer.

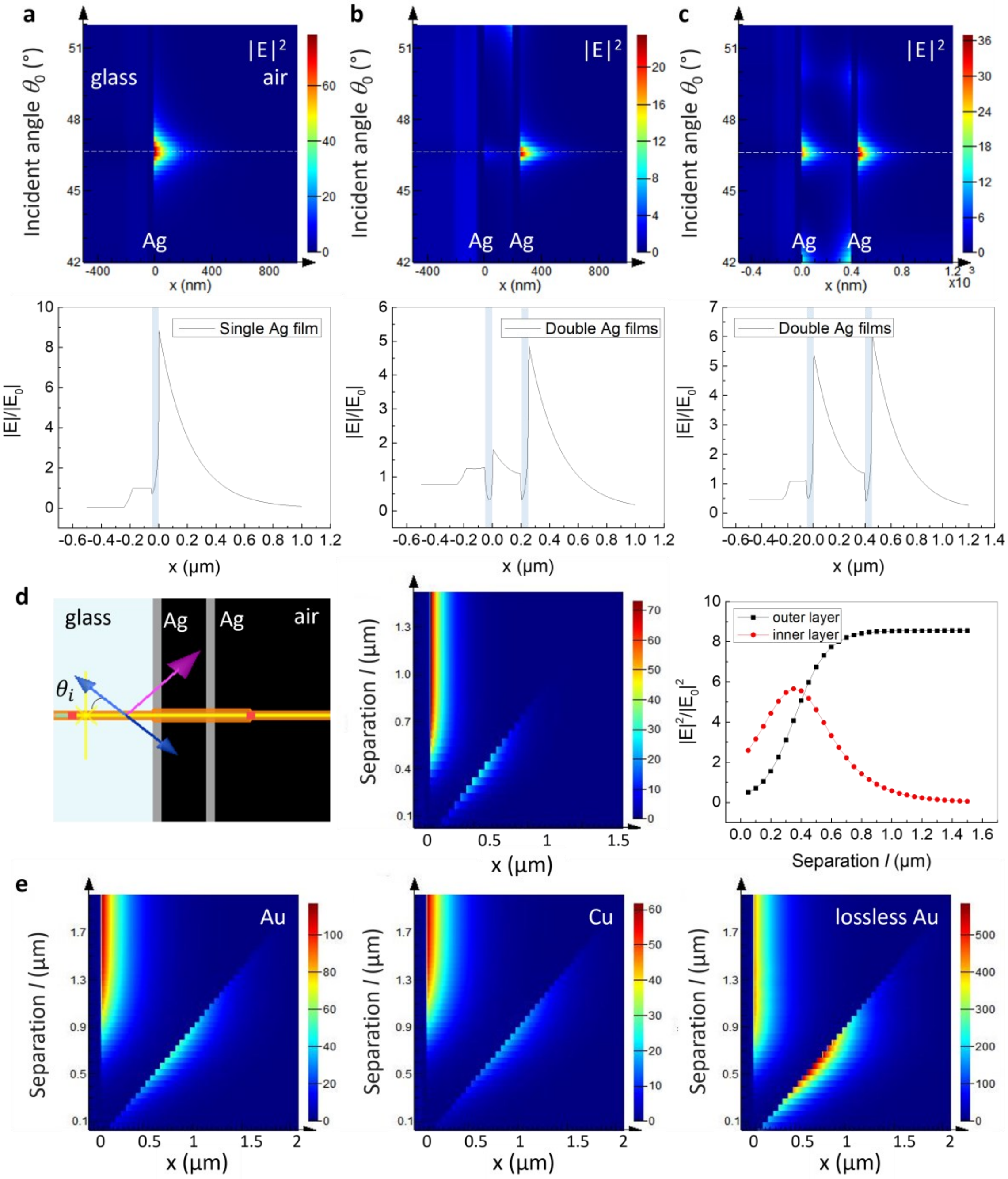

Supplementary Fig. 5. Evanescent near-field coupling as limited to submicrons in double metal thin films. (**a**) Surface plasmon polariton mode excited in a single-layer Ag film based on finite-difference time-domain (FDTD) simulation. Evanescent coupling between double Ag films with a separation distance of (**b**) l = 200 nm and, (**c**) l = 400 nm. Blue regions represent Ag thin films (thickness d = 50 nm) in the modeling. (**d**) Separation-dependent evanescent coupling between double Ag films. Different from ENZ thin films, evanescent wave coupling exists in double metal thin films, which sustain propagating surface plasmon polaritons. No correlated optical intensity oscillation was observed in double Ag thin films as a function of interlayer separation l. The near field enhancement is one order of magnitude weaker compared to the ENZ double layers. The coupling distance at submicron scales is also much shorter than

the ENZ thin films. Incident light was at TM polarization. Pump wavelength was at 500 nm, and the incident angle was θ_i = 46.8°. (**e**) Evanescent near-field coupling is observed in different metals, including Au, Cu, as well as lossless Au with a zero imaginary part of permittivity. The pump wavelength was at 700 nm. Compared to ENZ bilayers that show correlated phase shifts as controlled by the separation distance, propagating surface plasmon polaritons at the surface of two metal films have no correlated optical phases between each other. In addition, the near-field coupling through evanescent waves in metal films shows orders of magnitude shorter coupling distances, which are limited to submicron scales.

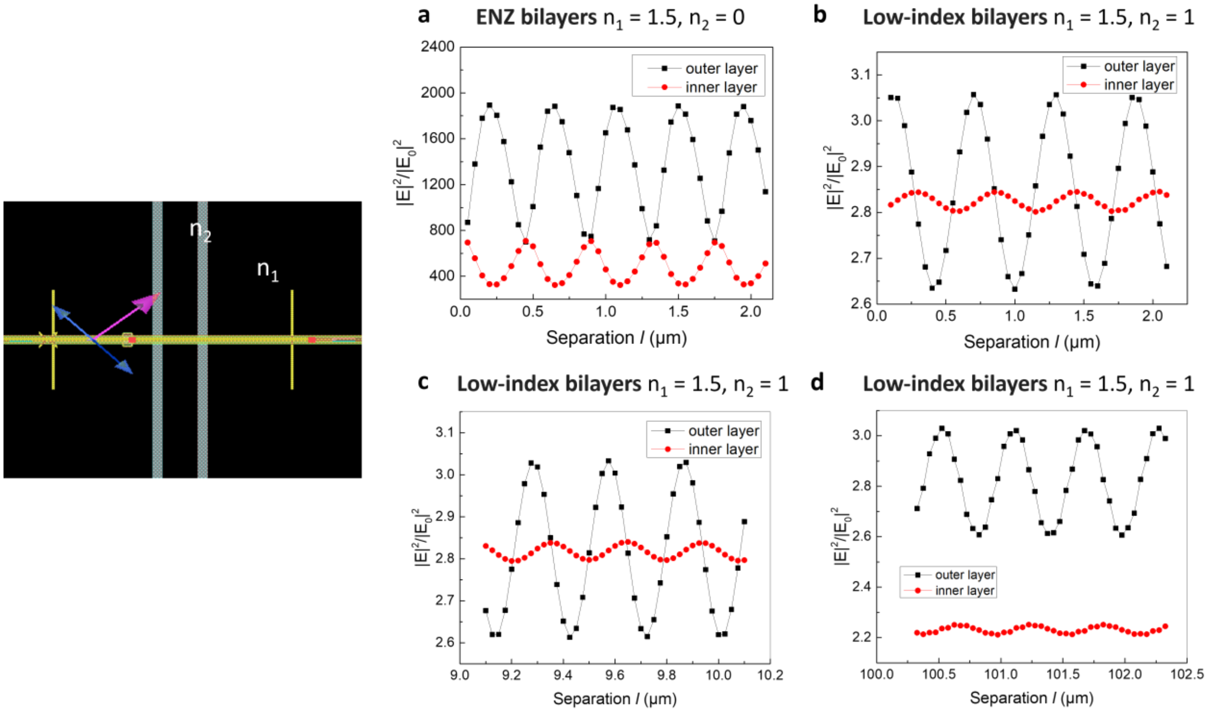

Supplementary Fig. 6. Comparison of near field oscillations between ENZ/silica/ENZ and air/silica/air multilayers. (**a**) Near-field oscillations for the ENZ/silica/ENZ system, where the near-field intensity is anti-correlated between the two ENZ layers. Incident angle was at θ_i = 7°. (**b**) Near-field oscillation for the air/silica/air dielectric system that exhibits weak optical interference. Incident angle was at θ_i = 42°, where the total internal reflection condition was met. (**c**) Near-field oscillations for the air/silica/air dielectric system at a separation l = 9.1 µm to 10.1 µm. (**d**) Near-field oscillations for the air/silica/air dielectric system at a separation l = 100.3 µm to 101.3 µm. Compared to ENZ double layers, uncorrelated near fields were observed between two low-index dielectric layers, where the optical fields are three orders of magnitude weaker. The weak thin-film interferences cannot sustain optical coupling with correlated near fields over distances. The background refractive index was n_1 = 1.5, and the pump wavelength was at λ = 1.3 µm for all panels. We compared the ENZ multilayer system to both metal and dielectric thin films that sustain leaky modes. Neither of these two cases showed correlated optical near fields, and both exhibited orders of magnitude shorter coupling distances compared to the ENZ multilayers.

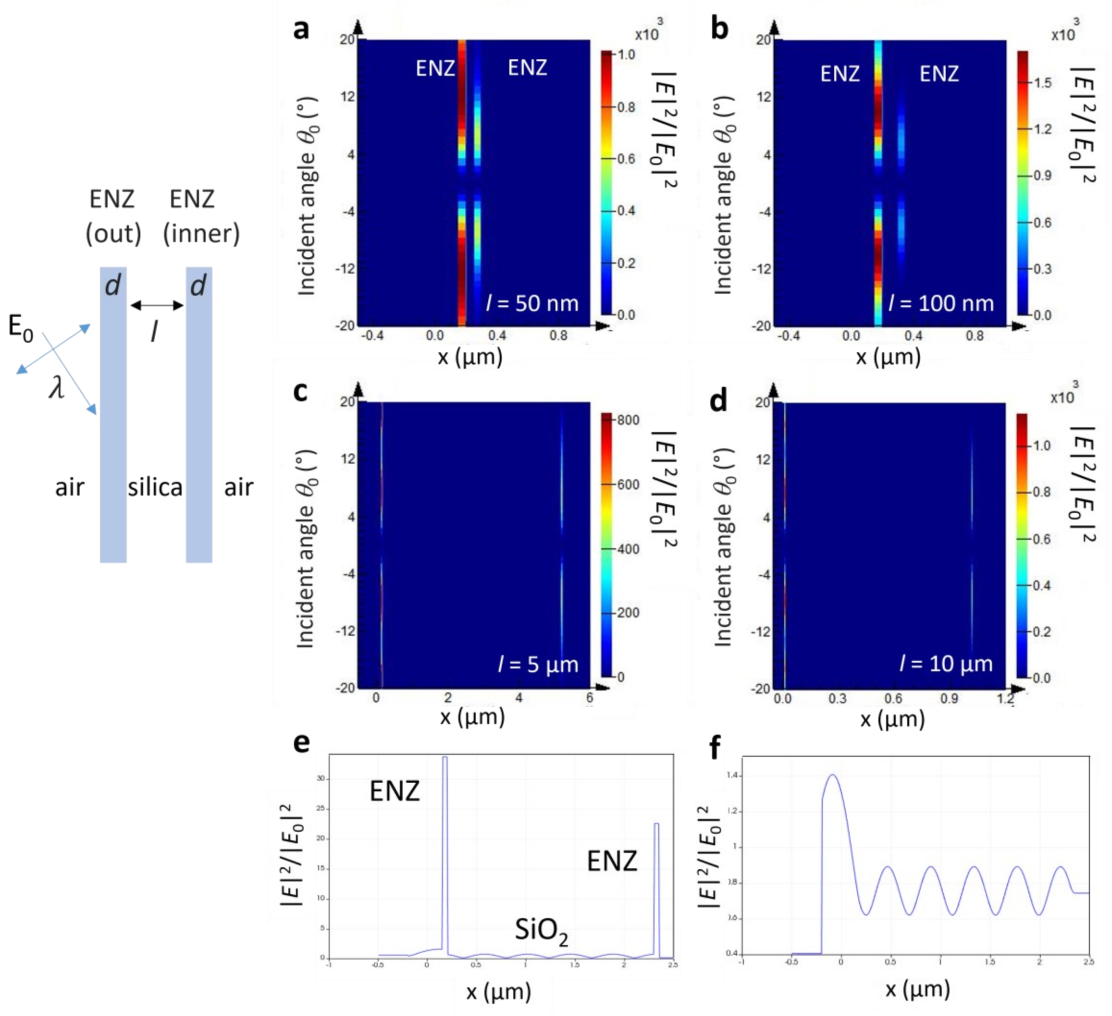

Supplementary Fig. 7. ENZ double-layers for long-distance optical coupling. Angle-resolved near field distributions for ENZ double layers with a silica spacer at a separation distance of (**a**) l = 50 nm, (**b**) l = 100 nm, (**c**) l = 5 µm, and (**d**) l = 10 µm. The optical coupling is sustained for ENZ double layers with varying interlayer separation l. (**e**-**f**) Numerical simulations showing the electric field distributions within the ITO and SiO_2 layer at ENZ resonance (panel e, TM polarization) and off resonance (panel f, TE polarization). As driven by the D field continuity at the materials interface under TM polarization, intense optical fields were observed within the ENZ layers in numerical simulations. In addition, electric field oscillations with characteristic standing wave patterns exist in the SiO_2 spacer layer, which is sandwiched by two ENZ layers.

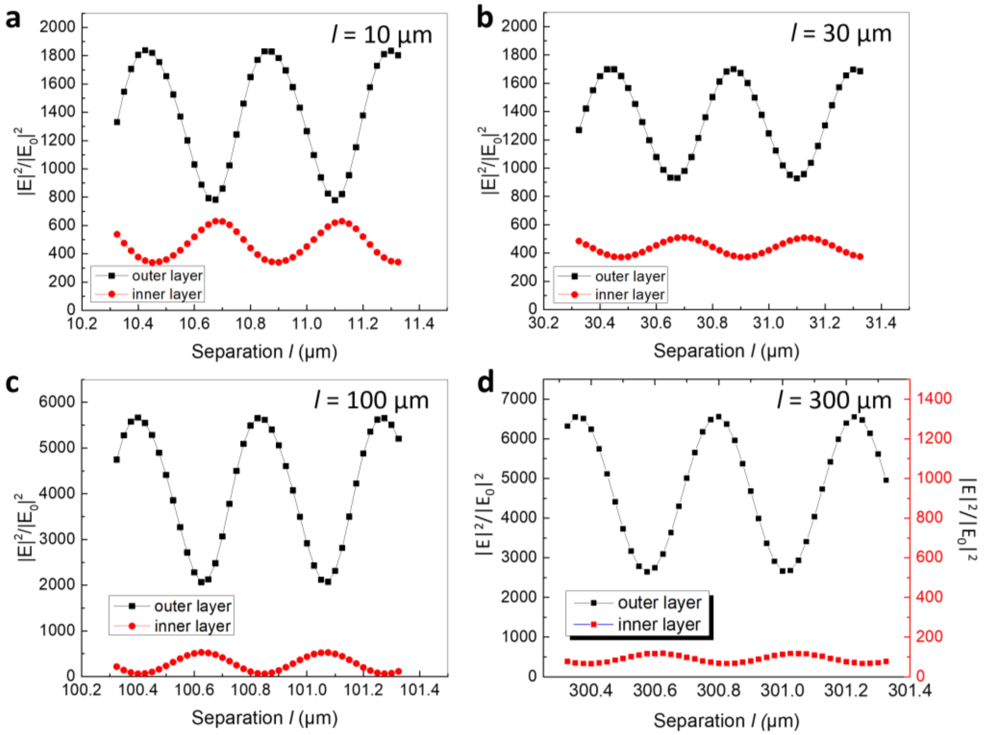

Supplementary Fig. 8. Long-distance coupling between ENZ double layers up to hundreds of microns with anti-correlated optical near fields. Modeled near field distributions and intensity oscillations for a separation between ENZ layers at (**a**) l = 10 µm, (**b**) l = 30 µm, (**c**) l = 100 µm, and (**d**) l = 300 µm. Pump wavelength is at λ = 1.3 µm for all panels. In FDTD modeling, the incident angle was at θ_i = 7° and ENZ double layers are on a silica substrate. The optical field intensities are anti-correlated between the outer and inner ENZ layers up to hundreds of microns.

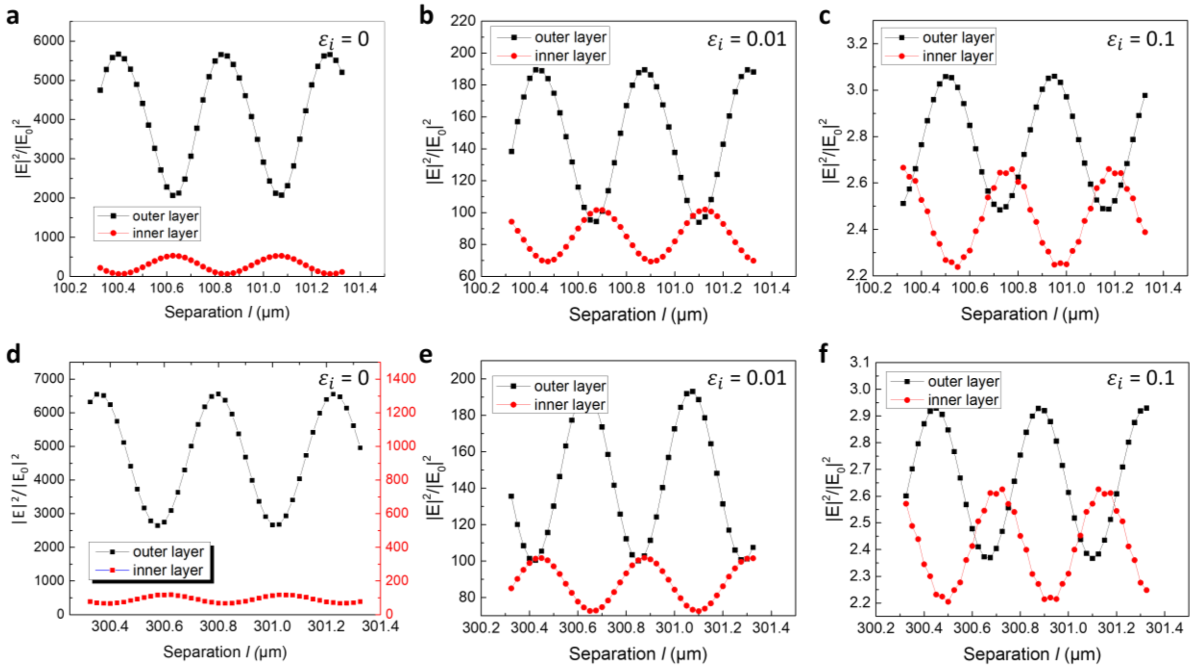

Supplementary Fig. 9. Sustained long-distance coupling up to hundreds of microns despite materials loss. Modeled near field distributions and intensity oscillations for an ENZ interlayer separation at l = 100 μm with materials loss (**a**) $\varepsilon_i = 0$, (**b**) $\varepsilon_i = 0.01$ and, (**c**) $\varepsilon_i = 0.1$, where ε_i is the imaginary part of permittivity. Modeled near field distributions and intensity oscillations for an ENZ interlayer separation at l = 300 μm with materials loss (**d**) $\varepsilon_i = 0$, (**e**) $\varepsilon_i = 0.01$ and, (**f**) $\varepsilon_i = 0.1$. The long-range coupling between ENZ layers is sustained up to a separation l = 300 μm despite materials loss in the modeling. Notably, previous literatures suggest potential enhancement of quantum tunneling despite a dissipative system[4,5].

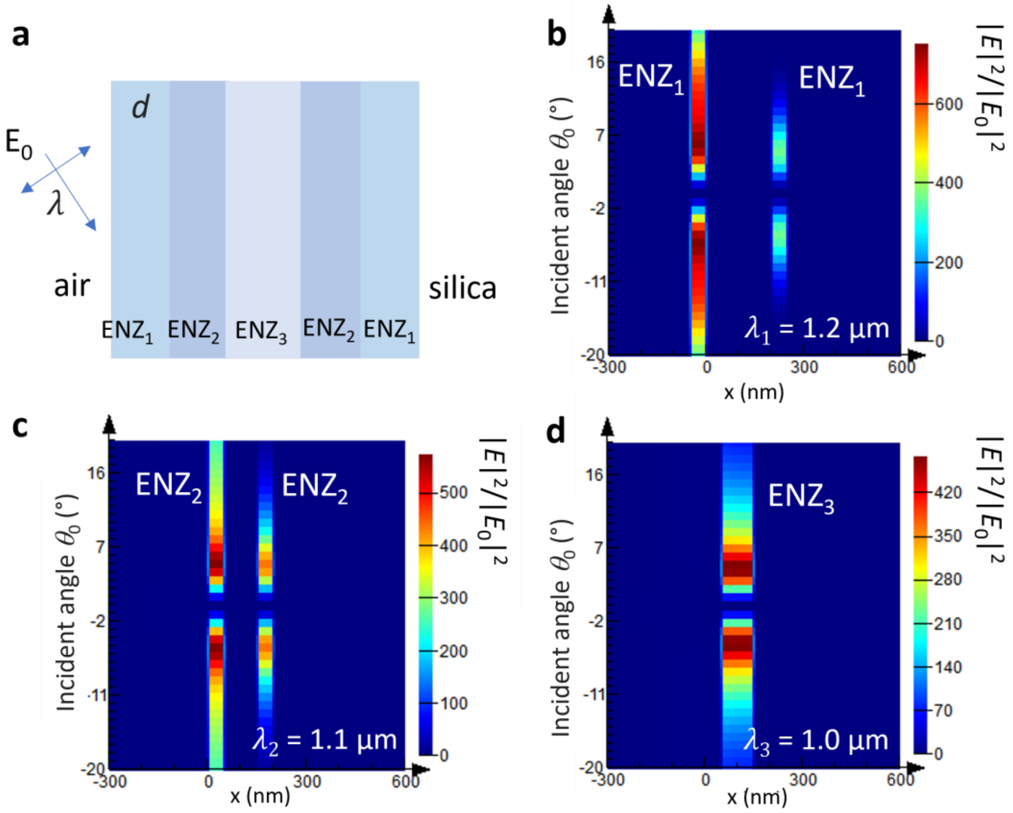

Supplementary Fig. 10. Wavelength as an additional degree of freedom in ENZ multilayers for spatial and spectral modulation of long-range optical coupling. (**a**) Scheme of ENZ multilayers containing three distinct ENZ wavelengths at 1.2 µm, 1.1 µm, and 1 µm, respectively, from left to right. Near-field distribution plots for ENZ multilayers at a pump wavelength of (**b**) $\lambda = 1.2$ µm, (**c**) $\lambda = 1.1$ µm and, (**d**) $\lambda = 1$ µm. ENZ film thickness is $d = 50$ nm. Here, varying the pump wavelength can selectively address the submicron regions of ENZ thin films that couple with each other.

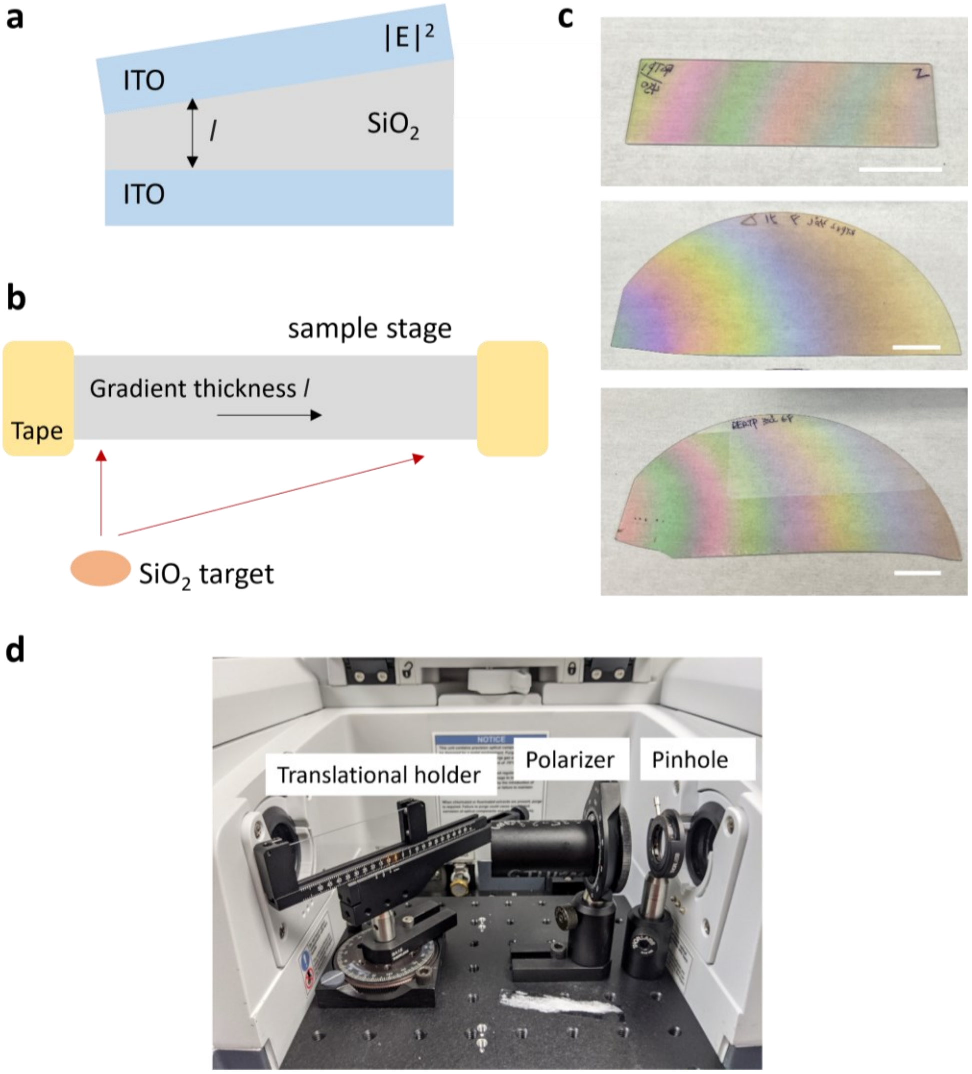

Supplementary Fig. 11. Angled sputtering deposition for fabricating wedged ITO thin films. (**a**) Scheme of the ITO multilayer structure with a wedged silica thin film in the middle for gradient change of the interlayer thickness. (**b**) Angled deposition process with AST sputter, where the sample holder is unrotated over sputtering deposition to produce a graded silica thin film with position-dependent thickness variation. (**c**) The images of fabricated ITO multilayers on a 3-inch glass slide and a 6-inch silica wafer, respectively. Inset scale bar is 2 cm. With a sputtering power of 100 W, Ar gas flow at 60 sccm, and deposition time up to 3 hours, the deposited SiO_2 layer showed a thickness variation from 100 nm to 800 nm. (**d**) Photo of the optical setup for measuring angle-resolved transmission spectra. A collimated, polarized circular beam goes through the ITO samples, where the spot position can be controlled by the translational holder with a 5 cm tuning range.

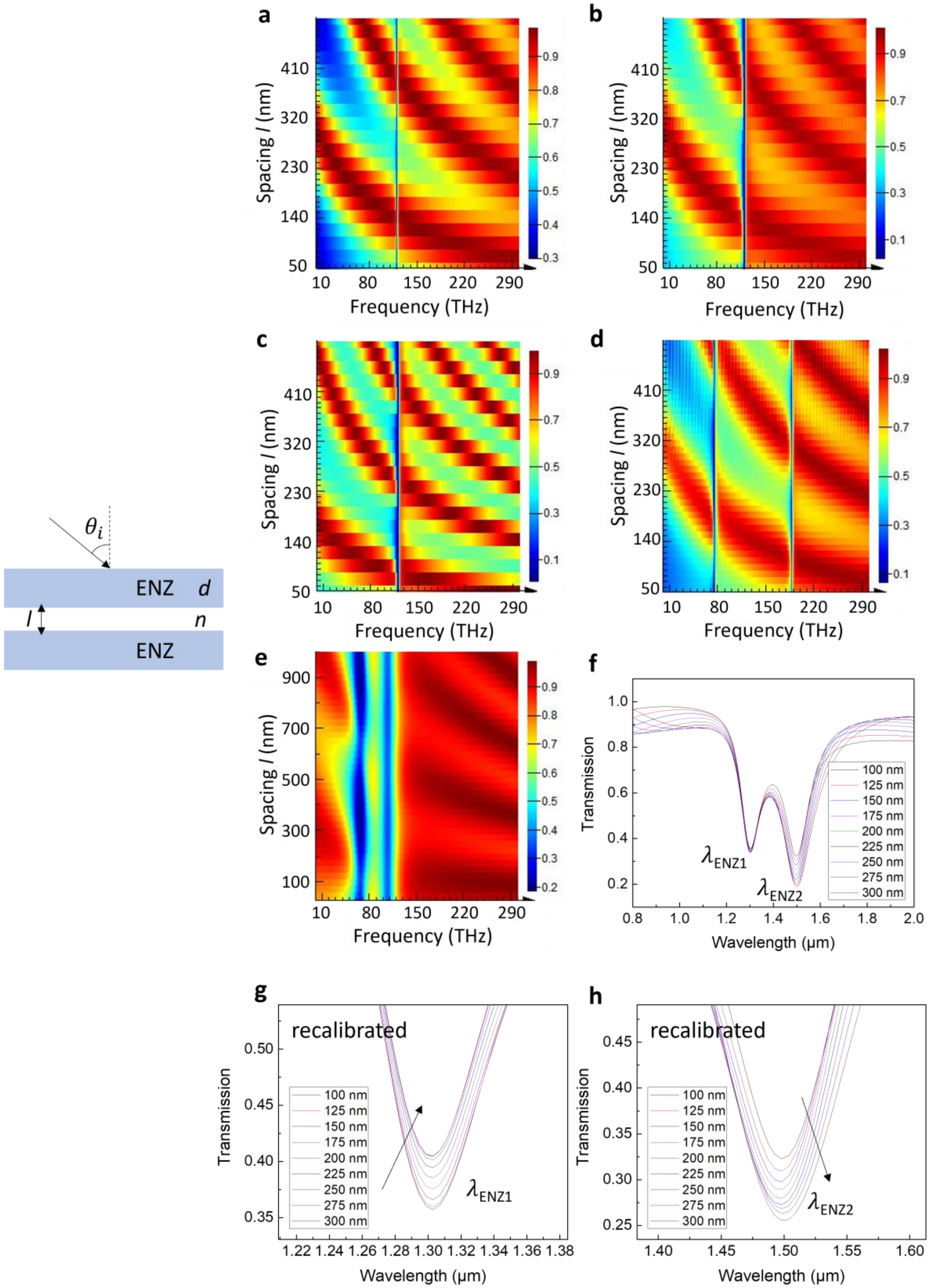

a
b
c
d
e
f
g
h
θ_i
ENZ
d
l
n
ENZ
Spacing l (nm)
Frequency (THz)
Transmission
Wavelength (μm)
λ_{ENZ1}
λ_{ENZ2}
recalibrated
100 nm
125 nm
150 nm
175 nm
200 nm
225 nm
250 nm
275 nm
300 nm

Supplementary Fig. 12. Separation-dependent transmission spectra showing the oscillatory resonance intensities modulated by interlayer ENZ coupling. The oscillatory transmission spectra for (**a**) spacer index n = 1 and incident angle θ_i = 2°, (**b**) n = 1 and θ_i = 5° and, (**c**) n = 1.5 and θ_i = 5° with an ENZ wavelength λ = 500 nm. Red curves represent the condition of destructive interference between two ENZ layers. The oscillatory transmission spectra for (**d**) n = 1 and θ_i = 5° with two distinct ENZ wavelengths at λ_1 = 400 nm and λ_2 = 600 nm and, (**e**) n = 1.5 and θ_i = 15° with two distinct ENZ wavelengths at λ_1 = 1300 nm and λ_2 = 1500 nm. (**f**) Modeled transmission spectra of double ENZ layers from panel e, where the resonance intensities at λ_1 and λ_2 are anti-correlated with each other. Such evolution of resonance intensity at λ_1 and λ_2 with increased spacer thickness agrees well with the measured transmission intensity changes in Figure 3b. (**g-h**) In order to better show the anti-correlated behavior between the resonance intensity at λ_{ENZ1} and λ_{ENZ2}, the transmission spectra in Figure S12f were replotted by calibrating to the background transmission intensity at 1.15 μm (l = 100 nm). The resonance intensity at λ_{ENZ1}, as quantified by the depth of the transmission dip, continuously decreased with an increased spacer separation from 100 nm to 300 nm. For λ_{ENZ2}, the resonance intensity continuously increased instead. Such evident anti-correlated behavior agrees well with the electric field intensity oscillation in Figure 2c. Note that the measured depth of the transmission dips at ENZ wavelengths is smaller than that in the modeling due to the material loss of ITO thin films.

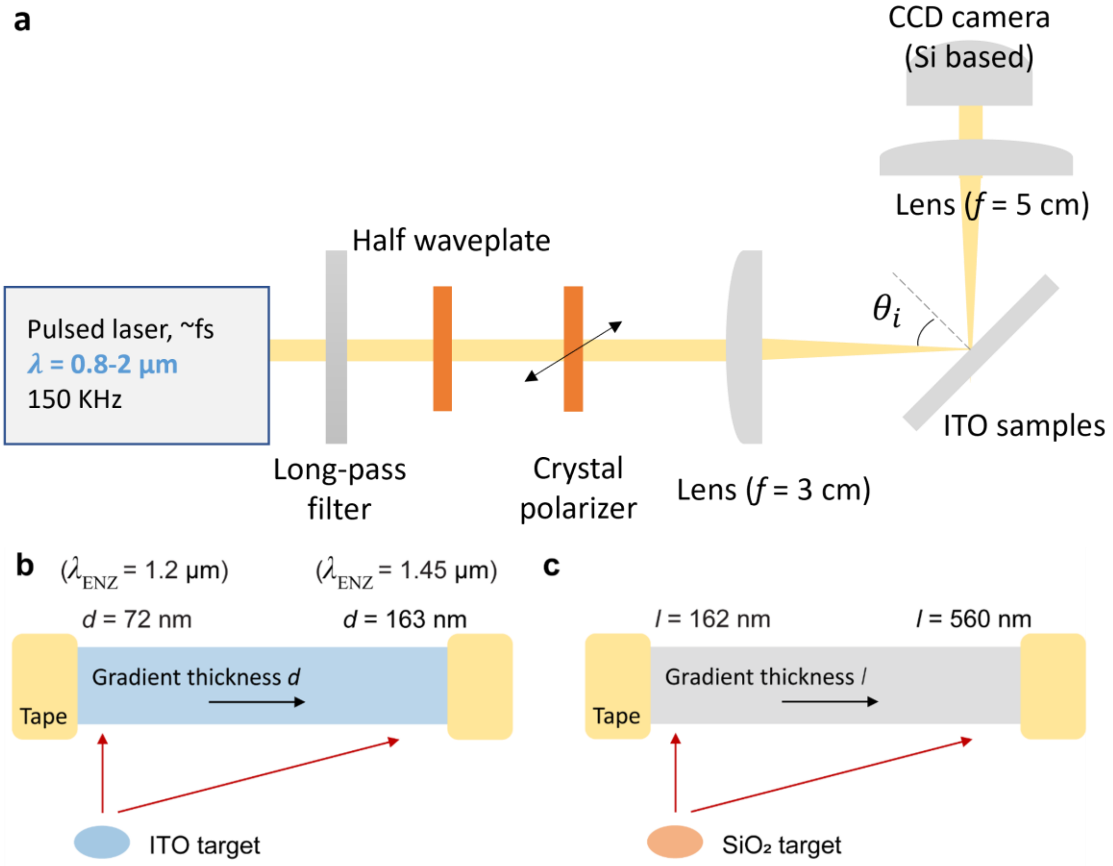

Supplementary Fig. 13. Scheme of the optical setup for nonlinear optical measurements on wedged ENZ multilayers at room temperature. (**a**) Optical setup for second harmonic generation (SHG) measurements. (**b**) Sample condition for the single-layer graded ITO thin film. (**c**) Sample condition for the ITO/SiO_2/ITO multilayers with a graded SiO_2 spacer. The ultrafast pulsed laser (~200 fs pulse length) has a modulation frequency of 150 kHz and a tunable emission output between 0.8-2 μm. The laser beam was focused by an optical lens (focal distance $f = 3$ cm) to the ITO sample surface with a 10-μm diameter spot and an incident angle of $\theta_i = 45°$. The emission signals at 45° were recollected by a focal lens ($f = 5$ cm) before entering a Si-based CCD camera. The condition of oblique incidence, transverse magnetic polarization, and a short-pulsed ~fs laser is critical for exciting SHG signals in ITO thin films.

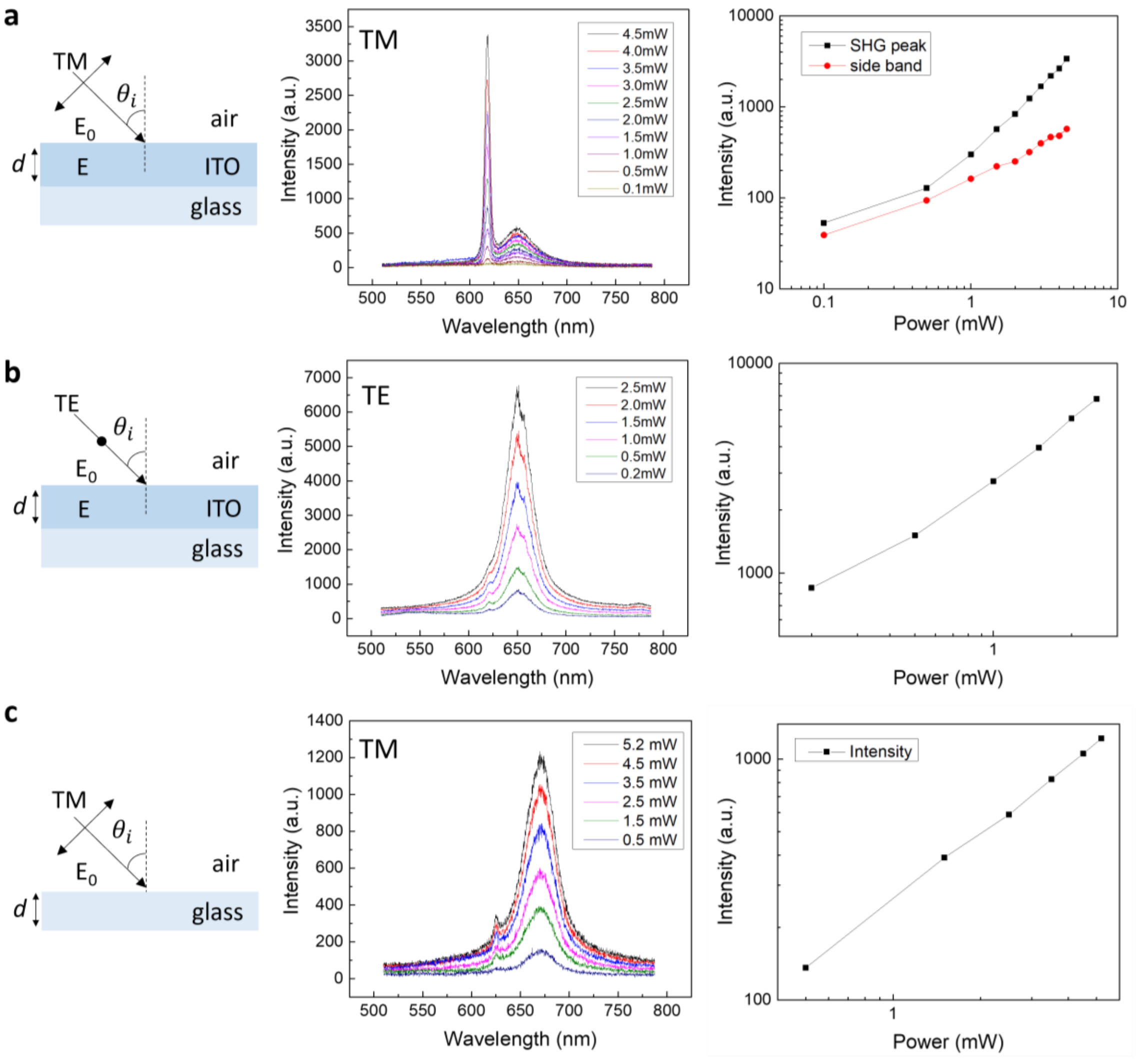

Supplementary Fig. 14. Measured SHG signals from a single-layer ITO thin film. (**a**) Power-dependent SHG output from an ITO thin film under TM polarization (power-law rising slope of 1.7) under a -fs pulsed pump and its comparison to the sideband signal from optical scattering (power-law rising slope of 0.98). (**b**) No SHG signal was observed from an ITO thin film under TE polarization. The side band showed linear dependence as a function of increased pump power, with a power-law rising slope of 0.99. (**c**) No SHG signal was observed from a glass substrate under TM polarization. The side band showed linear dependence as a function of increased pump power, with a power-law rising slope of 0.99. The pump wavelength is λ = 1.25 µm. The slightly deviated SHG peak position at 619 nm (from 625 nm) could come from minor optical misalignment in the light path. Enabled by the enhanced electromagnetic fields confined within ENZ thin films, strong SHG signals that scale with $|E|^4$ were observed from ITO thin films.

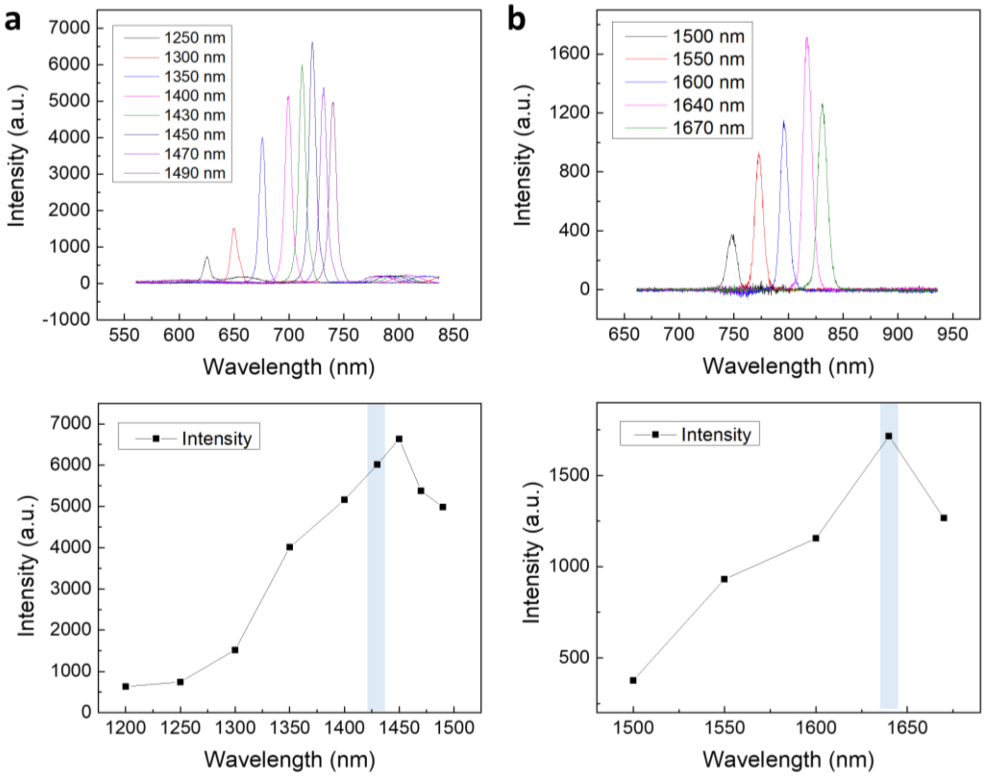

Supplementary Fig. 15. Modulated SHG intensity at ENZ wavelength in a single-layer ITO film. (**a**) Wavelength-dependent SHG signals measured from an ITO thin film with λ_{ENZ} = 1.43 µm. Pump power is at 2 mW with a beam spot diameter of ~10 µm. Pump wavelength λ_{pump} varies from 1250 nm to 1490 nm. (**b**) Wavelength-dependent SHG signals from an ITO thin film with λ_{ENZ} = 1.64 µm. Pump power is at 1 mW with a beam spot diameter of ~10 µm. Pump wavelength λ_{pump} varies from 1500 nm to 1670 nm. In panel a, a deviation of 20 nm was observed between the pump wavelength λ_{pump} that supports a maximum SHG intensity and the ENZ wavelength λ_{ENZ}, which can be attributed to a slightly different ENZ wavelength at the pump spot in the SHG measurement.

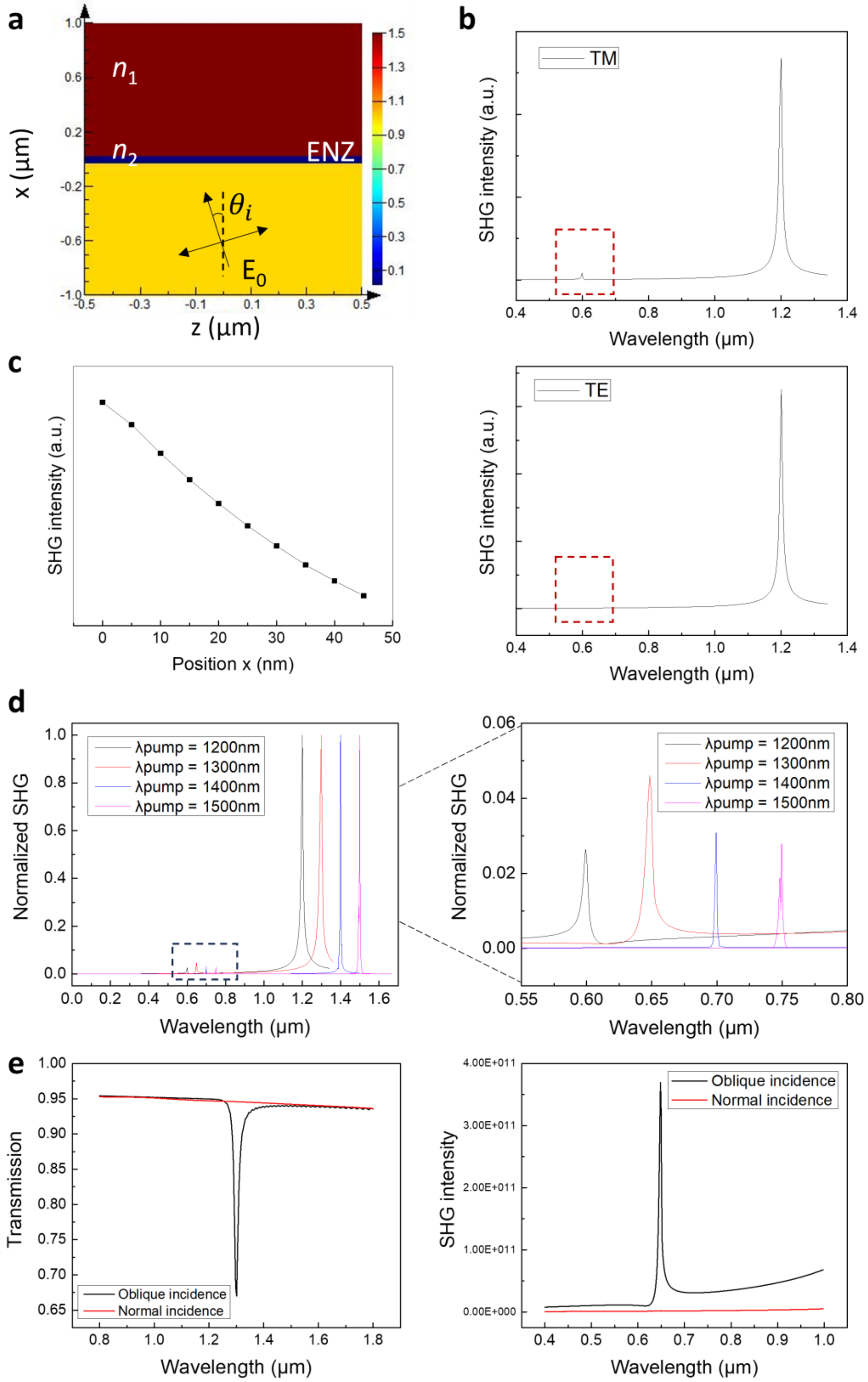
a
b
c
d
e
n_1
n_2
ENZ
θ_i
E_0
x (μm)
z (μm)
TM
TE
SHG intensity (a.u.)
Wavelength (μm)
Position x (nm)
Normalized SHG
λpump = 1200nm
λpump = 1300nm
λpump = 1400nm
λpump = 1500nm
Transmission
Oblique incidence
Normal incidence
SHG intensity

Supplementary Fig. 16. Modeled SHG signals from ENZ thin films based on the finite-difference time-domain method. (**a**) Scheme for modeling SHG from ENZ thin films. The optical pump is at λ = 1.2 μm with an oblique incident angle θ_i = 5°. (**b**) Modeled SHG responses at TM and TE polarization. The SHG intensity at 600 nm under TM polarization (dashed boxes) is two orders of magnitude stronger than that under TE polarization, the latter corresponding to the intrinsic nonlinear responses of the base ENZ material. (**c**) Spatial dependence of SHG intensity as a function of distance from the dielectric/ENZ interface. SHG is most intense at the dielectric/ENZ interface and decays exponentially within the ENZ thin film, consistent with the spatial distribution of electric field intensity in Supplementary Figs. 2e-f. (**d**) Modeling of the shifted SHG peaks from the ENZ thin film with a varying pump wavelength. (**e**) Modeling of ENZ resonance and SHG generation at oblique-angle incidence and normal incidence. As driven by the electric displacement field continuity at the materials interface ($\varepsilon_0 E_{1\perp} = \varepsilon_0 \varepsilon E_{2\perp}$), intense optical fields associated with a transmission dip at ENZ wavelength were observed at oblique angle incidence. In contrast, no ENZ resonance exists at normal incidence, and hence, the SHG signal was not observed.

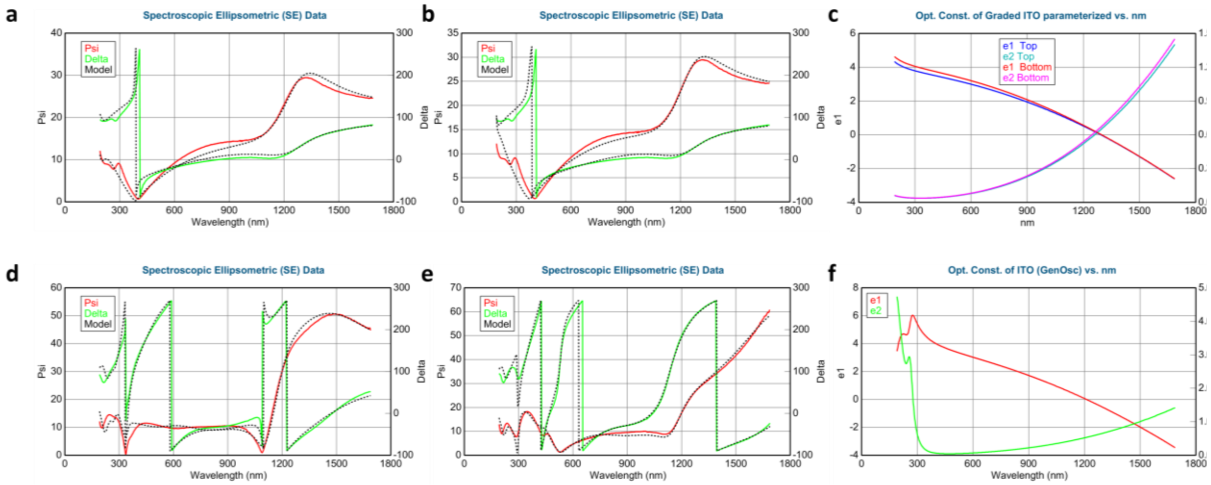

Supplementary Fig. 17. Fitting of ITO permittivity characterized by ellipsometry. (**a-c**) Optical permittivity of a single ITO thin film. (**d-f**) Optical permittivity of ITO/silica/ITO multilayers fabricated by magnetic sputtering, where the silica thickness varies from l = 133.5 nm to l = 214.5 nm. Solid lines represent the measured signals, and dashed lines show the ellipsometry fitting with a Drude model. The fitting results suggest no major difference in the permittivity with and without including a gradient refractive index distribution of ITO (panels a-b). The upper and lower layers of an ITO film show similar permittivity based on a graded ITO model (panel c). Using a homogeneous permittivity distribution for ITO thin films (thickness ~ 50 nm), the ellipsometry fitting model captured the varying silica film thickness in the ITO/silica/ITO multilayers (panels d-f).

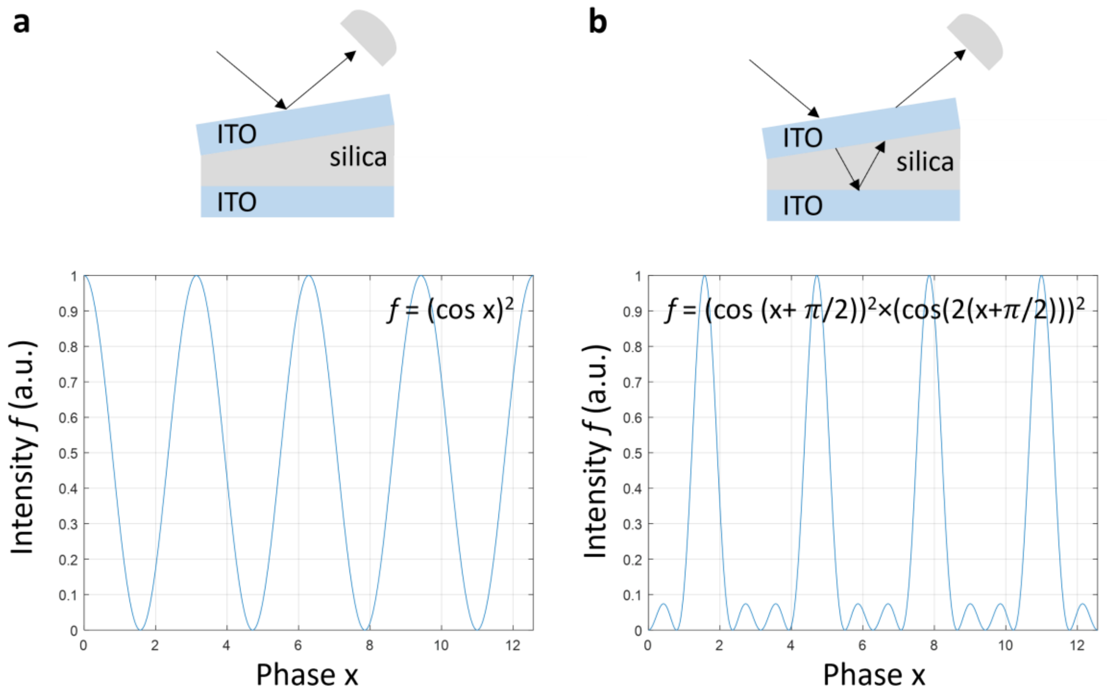

Supplementary Fig. 18. Mathematical model for modulated SHG signals from ITO multilayers. (**a**) SHG intensity from ITO double layers without additional interference at the ITO/silica/ITO interfaces. (**b**) SHG intensity from ITO double layers modulated by interference. In panel a, SHG signals from ITO multilayers showed an oscillatory intensity change governed by the oscillatory optical near fields. The SHG signals were further modulated by the interference at the ITO/silica/ITO interfaces, which led to a reduced oscillation periodicity and intensity change as consistent with measured SHG signals in Figs. 5c-d.

Supplementary References

1. Ferry, D. *Quantum mechanics: an introduction for device physicists and electrical engineers*. (CRC Press, 2020).
2. B. Ricco, M. Y. Azbel, Physics of Resonant Tunneling - the One-Dimensional Double-Barrier Case. *Phys. Rev. B* **29**, 1970-1981 (1984).
3. M. L. Strekalov, Quantum tunneling in an exactly solvable double-barrier potential: barrier transmission near the zero energy. *Journal of Mathematical Chemistry* **56**, 890-903 (2018).
4. K. Fujikawa, S. Iso, M. Sasaki, H. Suzuki, Quantum tunneling with dissipation: Possible enhancement by dissipative interactions. *Phys Rev B Condens Matter* **46**, 10295-10309 (1992).
5. H. Grabert, U. Weiss, P. Hanggi, Quantum tunneling in dissipative systems at finite temperatures. *Physical Review Letters* **52**, 2193 (1984).
