# Supplementary material for: Long-range optical coupling with epsilon-near-zero materials
Source: Nat Commun. 2025 Oct 16;16:9172. doi: 10.1038/s41467-025-64504-w (PMC12531341; doi:10.1038/s41467-025-64504-w)
Supplement: Supplementary file 2 — Description of Additional Supplementary Files [file 41467_2025_64504_MOESM2_ESM.pdf]

Description of Additional Supplementary Files

File Name: Supplementary Movie 1
Description: The modeled light propagation video ($|\mathbf{E}|^2/|\mathbf{E}_0|^2$) for a single-layer ENZ thin film with the input light under transverse magnetic polarization. The pump light is at ENZ wavelength λ_{pump} = 1.3 µm. The film thickness is d = 50 nm, and the incident angle is θ_i = 5°.

File Name: Supplementary Movie 2
Description: The modeled light propagation video ($|\mathbf{E}|^2/|\mathbf{E}_0|^2$) for a single-layer ENZ thin film with the input light under transverse electric polarization. The pump light is at ENZ wavelength λ_{pump} = 1.3 µm. The film thickness is d = 50 nm, and the incident angle is θ_i = 5°.

File Name: Supplementary Movie 3
Description: The modeled light propagation video ($|\mathbf{E}|^2/|\mathbf{E}_0|^2$) for double-layer ENZ thin films with the input light under transverse magnetic polarization. The pump light is at ENZ wavelength λ_{pump} = 1.3 µm. The film thickness is d = 50 nm, and the incident angle is θ_i = 5°.

File Name: Supplementary Movie 4
Description: The modeled light propagation video ($|\mathbf{E}|^2/|\mathbf{E}_0|^2$) for double-layer ENZ thin films with the input light under transverse electric polarization. The pump light is at ENZ wavelength λ_{pump} = 1.3 µm. The film thickness is d = 50 nm, and the incident angle is θ_i = 5°.
